# Metagenomic profiling of gut microbial communities in both wild and artificially reared Bar‐headed goose (*Anser indicus*)

**DOI:** 10.1002/mbo3.429

**Published:** 2016-12-20

**Authors:** Wen Wang, Sisi Zheng, Kirill Sharshov, Hao Sun, Fang Yang, Xuelian Wang, Laixing Li, Zhixiong Xiao

**Affiliations:** ^1^ Center of GrowthMetabolism and AgingKey Laboratory of Bio‐Resource and Eco‐Environment of Ministry of EducationCollege of Life Sciences and State Key Laboratory of BiotherapySichuan UniversityChengduChina; ^2^ Key Laboratory of Adaptation and Evolution of Plateau BiotaNorthwest Institute of Plateau BiologyChinese Academy of SciencesXi'ningChina; ^3^ University of Chinese Academy of SciencesBeijingChina; ^4^ Research Institute of Experimental and Clinical MedicineNovosibirskRussia

**Keywords:** Bar‐headed goose, gut microbiome, high‐throughput sequencing, metagenomics, taxonomic analysis

## Abstract

Bar‐headed goose (*Anser indicus*), a species endemic to Asia, has become one of the most popular species in recent years for rare bird breeding industries in several provinces of China. There has been no information on the gut metagenome configuration in both wild and artificially reared Bar‐headed geese, even though the importance of gut microbiome in vertebrate nutrient and energy metabolism, immune homeostasis and reproduction is widely acknowledged. In this study, metagenomic methods have been used to describe the microbial community structure and composition of functional genes associated with both wild and artificially reared Bar‐headed goose. Taxonomic analyses revealed that *Firmicutes*,* Proteobacteria*,* Actinobacteria* and *Bacteroidetes* were the four most abundant phyla in the gut of Bar‐headed geese. *Bacteroidetes* were significantly abundant in the artificially reared group compared to wild group. Through functional profiling, we found that artificially reared Bar‐headed geese had higher bacterial gene content related to carbohydrate transport and metabolism, energy metabolism and coenzyme transport, and metabolism. A comprehensive gene catalog of Bar‐headed geese metagenome was built, and the metabolism of carbohydrate, amino acid, nucleotide, and energy were found to be the four most abundant categories. These results create a baseline for future Bar‐headed goose microbiology research, and make an original contribution to the artificial rearing of this bird.

## 1 Introduction

The Bar‐headed goose (*Anser indicus*) is endemic to Asia, breeding in selected wetlands on the high plateaus of central Asia (Takekawa et al., 2012) and wintering in south‐central Tibet (Bishop, Song, Canjue, & Gu, 1997) and India (Javed et al., 2000). As one of the dominant waterfowl species in wetland areas in Qinghai‐Tibetan Plateau, Bar‐headed geese are increasingly being reared in several provinces of China for both conservation and economic development (Feare, Kato, & Thomas, 2010). However, Bar‐headed geese, even though easy to rear, display a reduced egg‐laying rate compared to wild geese, which limits the number of eggs available for artificial incubation. Thus far, there is no scientific research about the change in reproductive traits during early domestication of wild Bar‐headed geese. In recent years, the reproductive biology of the domestic geese has attracted increasing attention. Some differentially expressed genes relevant to the different reproductive stages (pre‐laying, egg‐laying, and broodiness) and different organs (hypothalamus, pituitary, and ovaries) were identified using high‐throughput next‐generation sequencing (Ding et al., 2014, 2015; Gao et al., 2015). However, the molecular mechanisms underlying poor reproductive performance in domestic geese remain poorly understood. It is therefore necessary to develop new strategies to solve the above problems.

For the past 10 years, gut microbial mutualisms, commensalisms, and pathogenic relationships have been considered to be an important factor for animal health and disease (Backhed, Ley, Sonnenburg, Peterson, & Gordon, 2005). Gut microbiota benefit the host in a variety of ways, such as regulating gut motility, modulating immune homeostasis (El Aidy, Dinan, & Cryan, 2014), absorbing nutrients (Kau, Ahern, Griffin, Goodman, & Gordon, 2011), producing vitamins, and metabolizing bile acids and sterols (O'Mahony, Clarke, Borre, Dinan, & Cryan, 2015). Results of extensive studies have also revealed the effects of gut microbiota on reproduction (Comninos, Jayasena, & Dhillo, 2014; Koren et al., 2012). It is well known that to successfully complete the sexual reproduction (including oviparity and viviparity) processes, many essential nutrients and energy are required for the formation and maturation of reproductive cells and the synthesis of a variety of proteins, hormones, and secretions (Sirotkin & Grossmann, 2015). Based on these findings, we propose a hypothesis that altered rearing patterns (wild vs. artificially reared) occurred at the early domestication of wild Bar‐headed geese, which altered the gut microbiota, resulting in a lower reproductive rate due to the dysregulation in nutrients and energy cycling. Therefore, to uncover the important functions of gut microbiome in domestic Bar‐headed geese reproduction and the underlying mechanisms, the first work is to figure out the differences in the gut microbiota of wild and artificially reared Bar‐headed geese.

Many studies on gut microbial community function have been conducted on some domestic bird species (e.g., chicken, turkey, duck, and ostrich) (Pan & Yu, 2014), however, only a very limited number of wild birds’ gut microbiome have been reported in the literature (reviewed by Kohl, 2012; Waite & Taylor, 2014, 2015; a series of studies such as Barbosa et al., 2016; Dewar et al., 2013; Dewar, Arnould, Krause, Dann, & Smith, 2014;). Our previous comparative study on the different Bar‐headed geese breeding patterns, using 16S rRNA sequencing has shown that marked differences in the gut microbiota existed between wild and artificially reared Bar‐headed geese (Wang et al., 2016). However, 16S rRNA sequencing data cannot provide further insight into the functional capabilities of these gut microbiota. Therefore, in this study, high‐throughput and sequence‐based comparative metagenomics were applied on the Illumina HiSeq 2500 platform (1) to investigate and to compare the gut microbiota associated with both wild and artificially reared Bar‐headed geese, and (2) to try to relate the functional genes of the gut microbial communities to the biological characteristics of this species. Combined, these data will enable a deeper exploration of the Bar‐headed geese gut microbiome, with the ultimate goal of developing rational strategies for improving the reproductive rate of artificially reared Bar‐headed geese.

## 2 Materials and Methods

### 2.1 Ethics statement

This study was carried out in strict accordance with the Animal Management Rule of the National Health and Family Planning Commission, People's Republic of China (Documentation 55, 2001). The research protocol was reviewed and approved by the Animal Care and Use Committee of the Chinese Academy of Sciences. Fecal samples collection was authorized by the government officer from the Administration for Wildlife Protection, Qinghai Provincial Department of Forestry.

### 2.2 Fecal samples collection

Fecal samples from two groups were obtained in Qinghai province, China, in July, 2014. Two fecal samples were collected from two individual wild Bar‐headed geese from Ha Datan wetland (37°07′41.3″ N, 99°43′39.9″ E, elevation 3,100 m) nearing Bird Island of Qinghai Lake. Similarly, two samples from 2 individual artificially reared (abbreviation: AR) Bar‐headed geese were derived from Bu Ha river estuary (36°58′25.5″ N, 99°50′19.2″ E, elevation 3,197 m) in Qinghai Lake. The AR populations were not treated with antibiotics and raised from artificial incubation of wild Bar‐headed geese eggs. As a herbivorous bird, the nourishment of wild populations is composed of highly fibrous plant material, mainly grass, leaves, twigs, and seeds (Middleton & Ag, 1987). The AR populations had unrestricted access to fly away to seek natural food and were also fed on artificial diets (blends of 60% corn flour, 20% soybean flour, and vegetables). All four birds were adults, but their exact ages were unknown. About 1 g of fecal sample was collected from fecal balls, avoiding collection of fecal material that was touching the ground. All samples were placed in sterile containers and transported to the laboratory in a car‐carried refrigerator. In the laboratory, fecal samples were kept frozen at −80°C until processing.

### 2.3 DNA extraction and shotgun metagenomic sequencing

Genomic DNA was isolated from approximately 1 g of fecal sample, using the E.Z.N.A. ^®^ stool DNA Kit (Omega Bio‐tek, Norcross, GA, USA) following the manufacturer's instruction. DNA concentration and quality were determined, using QuantiFluor™ ‐ ST (Promega, Madison City, WI, USA) and gel electrophoresis, respectively. With the extracted DNA, library construction was performed on an Illumina Hiseq2500 platform according to the standard protocols. Metagenome sequences data are now available at NCBI under the Sequence Read Archive (SRA) database with accession no.SRP072790 and no.SRP072793.

### 2.4 Bioinformatic analysis of sequencing data

Raw sequences obtained from 4 metagenomic samples were subjected to a quality check, using the FastQC software (version v0.11.3) (Andrews, 2012). All samples showed satisfactory values for each parameter tested. Next, the sequences were run through Trimmomatic (version 0.33) (Bolger, Marc, & Bjoern, 2014) to remove low‐quality base pairs, using these parameters (SLIDINGWINDOW: 4:15 MINLEN: 36). Further, the host specific and other eukaryotic sequences were removed by parsing the NCBI nonredundant protein database (NCBI‐nr) taxonomic assignment using the lowest common ancestor (LCA) algorithm in MEGAN (Huson, Auch, Qi, & Schuster, 2007). The resulting cleaned sequences were analyzed by LCA algorithm in MEGAN to identify bacterial taxa, and were also analyzed by DIAMOND (version 0.7.9) (Buchfink, Xie, & Huson, 2015) against the NCBI‐nr, COG (Powell et al., 2012) and the KEGG (Kanehisa & Goto, 2000) databases to identify functional groups.

### 2.5 Metagenomic assembly and gene prediction

All the cleaned sequences obtained from four samples were mixed together and assembled de novo, using MEGAHIT (version 1.0.2) (Li, Liu, Luo, Sadakane, & Lam, 2015). From the resulting contigs, microbial genes were predicted using Prodigal (version 2.6.2) (Hyatt, LoCascio, Hauser, & Uberbacher, 2012). The function assignment of all ORFs were conducted, using DIAMOND (version 0.7.9) and BLASTX (version 2.2.31+) against COG and KEGG databases.

### 2.6 Statistical analysis

Two‐sided Welch's t‐test in STAMP software package was applied to test the differences between AR and Wild group (Parks & Beiko, 2010). A *p* value of <.05 were considered to be significant. All statistics and graphics were performed using customized R scripts.

## 3 Results

### 3.1 Summary of sequencing data

Illumina sequencing for all the fecal samples was performed, using a HiSeq2500 instrument (one lane, paired ‐ end run (2 × 125 bases)). The output data encompassed a total of 0.17 billion raw reads comprised of 21.78 billion bases (Table S1). From these reads, a total of 160 million reads were generated after applying strict trimming and filtering criteria to exclude low‐ reads, and the reads average length was 120 bases (Table S1). To avoid introducing any eukaryotic sequences into our dataset, we mapped all these quality passed reads to NCBI nonredundant database, and finally removed the representative eukaryotic genome (*Bovinae*,* Ovis*, and *Babesia bigemina*) presented in all the fecal samples. As a result, a total of 57.3 million clean reads were used (Table S2) for further clear assembly and annotation analysis.

### 3.2 Taxonomic compositions of the Bar‐headed geese gut microbial communities

**Figure 1:**
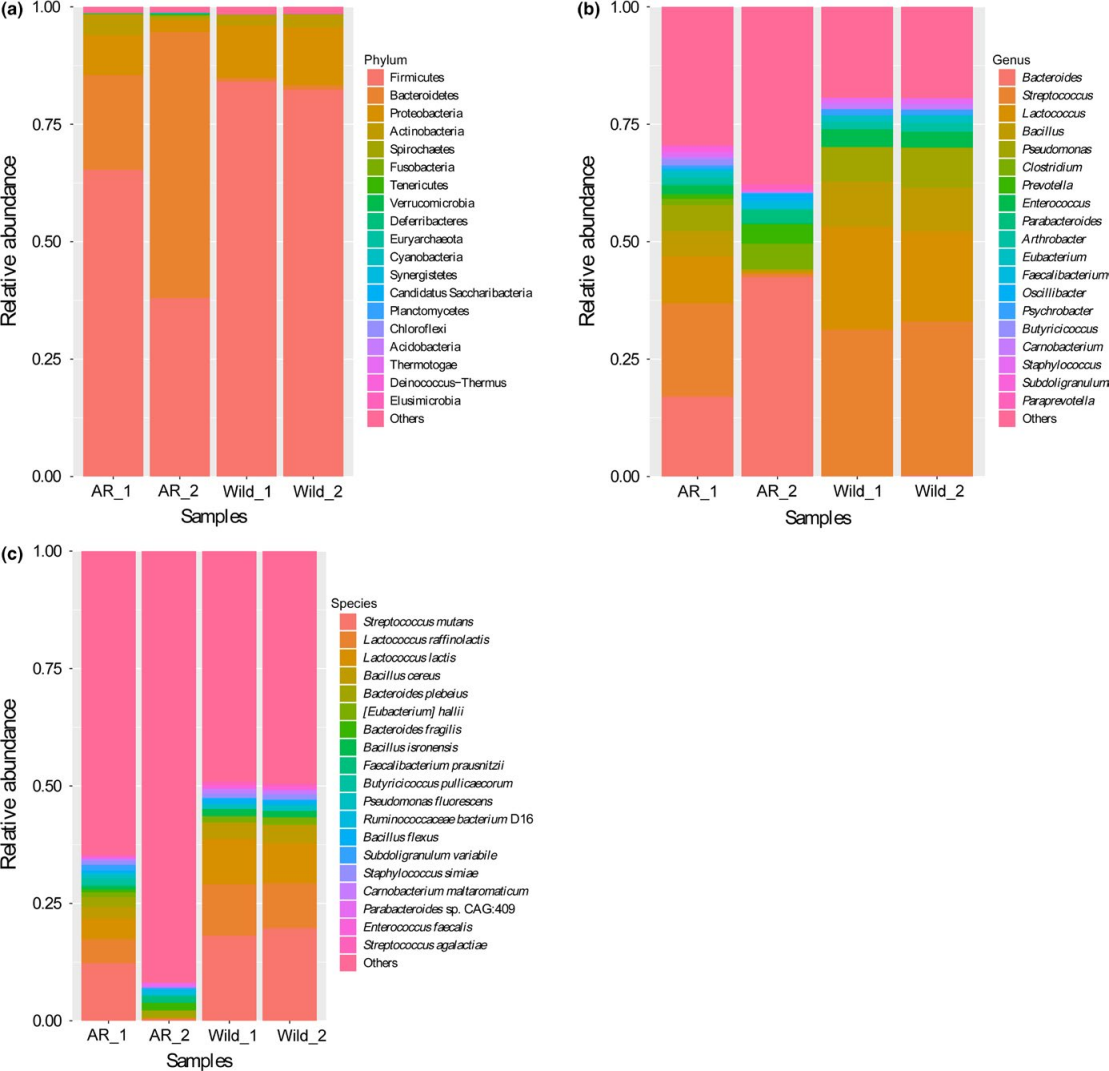
Taxonomic classifications of each sample at the (a) phylum, (b) genus and (c) species level

Results of the MEGAN analysis revealed a diverse gut microbial community in both wild and artificially reared Bar‐headed geese (Figure 1). For the wild group (Figure 1a), the predominant phylum in the microbial metagenome was the phylum *Firmicutes*, with an average relative abundance of 83.20%. The second predominant bacterial lineage, constituting 11.76%, was identified as phylum *Proteobacteria* and was followed by *Actinobacteria* and *Bacteroidetes*, accounting, respectively, for 2.48% and 0.86% relative abundance. In the AR group, *Firmicutes* also held the overwhelming predominance, with the average relative abundance of 51.63%, followed by *Bacteroidetes* (38.41%), *Proteobacteria* (5.52%), and *Actinobacteria* (2.49%). The additive abundance of these four most dominant phyla, was above 98% across all the samples. Phylum‐level comparative analyses showed that *Bacteroidetes* abundances tended to increase in AR group.

At the genus level, the sequences from the wild samples represented 106 genera and 191 different genera in the AR samples. The top 19 genera were listed in Figure 1b. We further found that the top seven abundant genera contributed between 44.10% and 75.02% of the total microbial abundance in both AR and wild samples (Table 1). These seven genera were distributed in the above‐described four dominant phyla (Table 1). Among them, four genera (*Streptococcus*,* Lactococcus*,* Bacillus*, and *Enterococcus*) belonged to *Firmicutes*, while for other three phyla, each containing one genus, such as *Proteobacteria* (genus *Pseudomonas*), *Actinobacteria* (genus *Arthrobacter*), and *Bacteroidetes* (genus *Bacteroides*) (Table 1). Genus‐level comparative analyses showed that *Bacteroides* abundances tended to increase in the AR group, while other six genera were all decreased in this group.

**Table 1:** The top 7 most abundant genera (%) in each group

Phylum	Genus	Wild group	AR group
Firmicutes	Streptococcus	31.825	10.165
Firmicutes	Lactococcus	20.465	5.190
Firmicutes	Bacillus	9.450	2.895
Proteobacteria	Pseudomonas	7.750	2.830
Firmicutes	Enterococcus	3.535	0.975
Actinobacteria	Arthrobacter	1.715	0.700
Bacteroidetes	Bacteroides	0.135	29.580
	Others	21.395	10.600

The relative abundances of each species were estimated from the number of assigned sequences with the lowest common ancestor (LCA) algorithm, around 434 species and about 240 species were found in the artificially reared and wild group, respectively. The top 19 species were listed in Figure 1c. The majority of species‐level phylotypes occurred at low levels, whereas the unclassified species accounted for large proportions, which ranged from 36.63% to 66.93% among the different samples (Table 2). Among these top 19 species, seven species (including *Bacteroides fragilis*,* Faecalibacterium prausnitzii*,* Butyricicoccus pullicaecorum*,* Ruminococcaceae bacterium D16*,* Subdoligranulum variabile*, and the *Parabacteroides sp. CAG:409* and *Bacteroides plebeius*) were not present above the 0.1% abundance level in any of the wild samples, but increased to an average cumulative abundance of 0.7% in AR samples (Table 2). In contrast, wild samples contained high abundance levels of the remaining 12 species (Table 2): *Streptococcus mutans*,* Lactococcus raffinolactis*,* Lactococcus lactis*,* Bacillus cereus*,* [Eubacterium] hallii*,* Bacillus isronensis*,* Pseudomonas fluorescens*,* Bacillus flexus*,* Staphylococcus simiae*,* Carnobacterium maltaromaticum*,* Enterococcus faecalis*, and *Streptococcus agalactiae*. The distributions of these species generally followed the pattern observed for genera, with species of *Bacteroides* abundant in AR group and species of genera *Streptococcus*,* Lactococcus*,* Bacillus*,* Enterococcus*, and *Pseudomonas* increased in wild group.

**Table 2:** The top 19 most abundant species (%) in each group

Phylum	Genus	Species	Wild group	AR group
		Others	37.09	57.98
Firmicutes	Streptococcus	Streptococcus mutans	18.61	6.12
Firmicutes	Lactococcus	Lactococcus raffinolactis	10.15	2.60
Firmicutes	Lactococcus	Lactococcus lactis	8.91	2.24
Firmicutes	Bacillus	Bacillus cereus	3.69	1.26
Firmicutes	Eubacterium	[Eubacterium] hallii	1.44	0.54
Firmicutes	Bacillus	Bacillus isronensis	1.44	0.36
Proteobacteria	Pseudomonas	Pseudomonas fluorescens	1.18	0.44
Firmicutes	Bacillus	Bacillus flexus	1.12	0.28
Firmicutes	Staphylococcus	Staphylococcus simiae	1.04	0.43
Firmicutes	Carnobacterium	Carnobacterium maltaromaticum	0.98	0.24
Firmicutes	Enterococcus	Enterococcus faecalis	0.73	0.21
Firmicutes	Streptococcus	Streptococcus agalactiae	0.72	0.19
Bacteroidetes	Bacteroides	Bacteroides fragilis	0.01	1.03
Firmicutes	Faecalibacterium	Faecalibacterium prausnitzii	0.01	0.86
Firmicutes	Butyricicoccus	Butyricicoccus pullicaecorum	0.00	0.75
Firmicutes	Unclassified	Ruminococcaceae bacterium D16	0.01	0.75
Firmicutes	Subdoligranulum	Subdoligranulum variabile	0.00	0.74
Bacteroidetes	Parabacteroides	Parabacteroides sp. CAG:409	0.00	0.49
Bacteroidetes	Bacteroides	Bacteroides plebeius	0.00	0.31

### 3.3 Functional analysis of the Bar‐headed geese gut microbiome

To investigate functional differences in the gut microbiota between the two groups, we performed functional profile analyses based on clean shotgun sequencing reads, using database of orthologous gene groups (COG and KEGG). The functional information of these reads was compared against COG database and the KEGG database, and 35.22%–44.90% and 23.18%–30.55% of which were, respectively, identified as COG and KEGG genes (Table S3).

**Figure 2:**
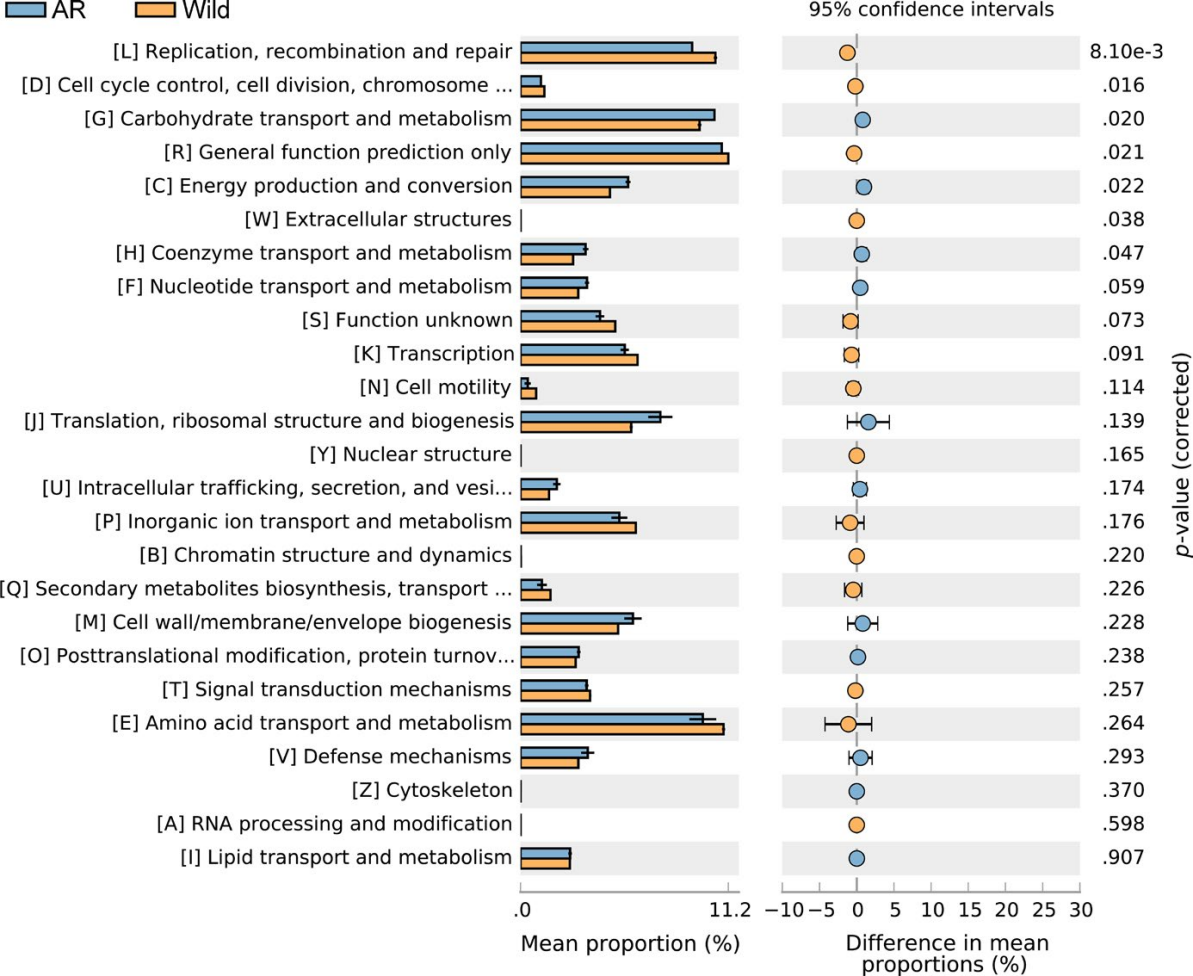
Significant COG categories differences between the AR and Wild metagenomes conducted with the STAMP program. Bars on the left represent the proportion of each category in the data. Categories difference with a *p* value of <.05 were considered to be significant

To determine biologically significant differences, the 25 functional COG categories detected in the AR group were statistically compared with the wild group (Figure 2). Comparison revealed a high degree of similarity between the two groups. However, some differences were observed with significantly over abundant reads in the AR group, which were assigned to carbohydrate transport and metabolism (COG category [G]), energy metabolism (COG category [C]) and coenzyme transport and metabolism (COG category [H]). By contrast, the wild group identified more reads in cell‐cycle control, cell division, chromosome partitioning (COG category [D]), replication, recombination and repair (COG category [L]), and extracellular structures (COG category [W]). A proportion of 15.76% of sequences were assigned to the COG category [S] (function unknown) and COG category [R] (General function prediction only), indicating a small number of potential unknown functional genes in Bar‐headed geese gut microbiota. Based on top BLASTX hits, the top 10 COG genes enriched in phylum *Bacteroidetes* in the AR group were shown in Table S4. Interestingly, these 10 genes were distributed in *Firmicutes* and *Proteobacteria* in the wild group due to the very low abundance of *Bacteroidetes* in this group.

Furthermore, we determined changes in functional composition, using the KEGG hierarchical classification at the KEGG orthologous group (KOs) and the pathway level. Among the detected KEGG pathways, “Protein digestion and absorption” [PATH: ko04974], “Glycosphingolipid biosynthesis ‐ ganglio series” [PATH: ko00604] and “Lysosome” [PATH: ko04142] were found to be the most significantly abundant ones in the AR group compared to the wild group. Interestingly, 9 KOs, mapped to these 3 KEGG pathways, respectively, were found to be significantly higher in AR group compared to wild group (Table 3). Additionally, taxonomic assignment of all these 9 KOs belonged to *Bacteroidetes*.

**Table 3:** The top three enriched KEGG pathways and corresponding top BLASTX hit organisms in the AR group

Pathway	Name	KOs significantly abundant in AR group	Top BLASTX hit organism in AR group
ko04974	Protein digestion and absorption	K01278, dipeptidyl‐peptidase 4 [EC:3.4.14.5]	Bacteroidetes (98.84%)
ko00604	Glycosphingolipid biosynthesis – ganglio series	K12373, hexosaminidase [EC:3.2.1.52]	Bacteroidetes (92.84%)
ko04142	Lysosome	K01201, glucosylceramidase [EC:3.2.1.45]	Bacteroidetes (75.09%)
		K01205, α, N‐acetylglucosaminidase [EC:3.2.1.50]	Bacteroidetes (99.29%)
		K01195, beta‐glucuronidase [EC:3.2.1.31]	Bacteroidetes (42.57%)
		K12373, hexosaminidase [EC:3.2.1.52]	Bacteroidetes (92.84%)
		K01192, beta‐mannosidase [EC:3.2.1.25]	Bacteroidetes (73.63%)
		K01186, sialidase‐1 [EC:3.2.1.18]	Bacteroidetes (85.21%)
		K01134, arylsulfatase A [EC:3.1.6.8]	Bacteroidetes (92.09%)

### 3.4 De novo assembly, gene prediction, and functional annotation

To create a one global gene catalog in the Bar‐headed geese metagenome, we performed de novo assembly of all the clean reads aforementioned (Table S2) for a pool, resulting in a total of 458,044,019 bases, with an N50 of 1,306 bp (389,629 contigs range from 500 bp to 209,039 bp). For gene prediction, 714,434 coding sequences (CDS) were identified, using the program Prodigal. All putative protein coding sequences were searched against the databases COG and KEGG, and the results were summarized in Table 4.

**Table 4:** Metagenome assembly statistics

Assembly metric	Our assembly
Total size	458,044,019 bp
Number of contigs	389,629
N50 value	1,306 bp
Largest contig	209,039 bp
GC (%)	45.48
Predicted genes	714,434
Match in KEGG Genes	550,830
Of these, assigned KO	289,467
Unique KOs	5,003
Match in KEGG pathways	357,857
KEGG pathways	262
Match in COG Genes	507,204
Of these, assigned COG	428,274
Unique COGs	3,820

### 3.5 General analysis of the metabolic potential encoded by the Bar‐headed geese metagenome

**Figure 3:**
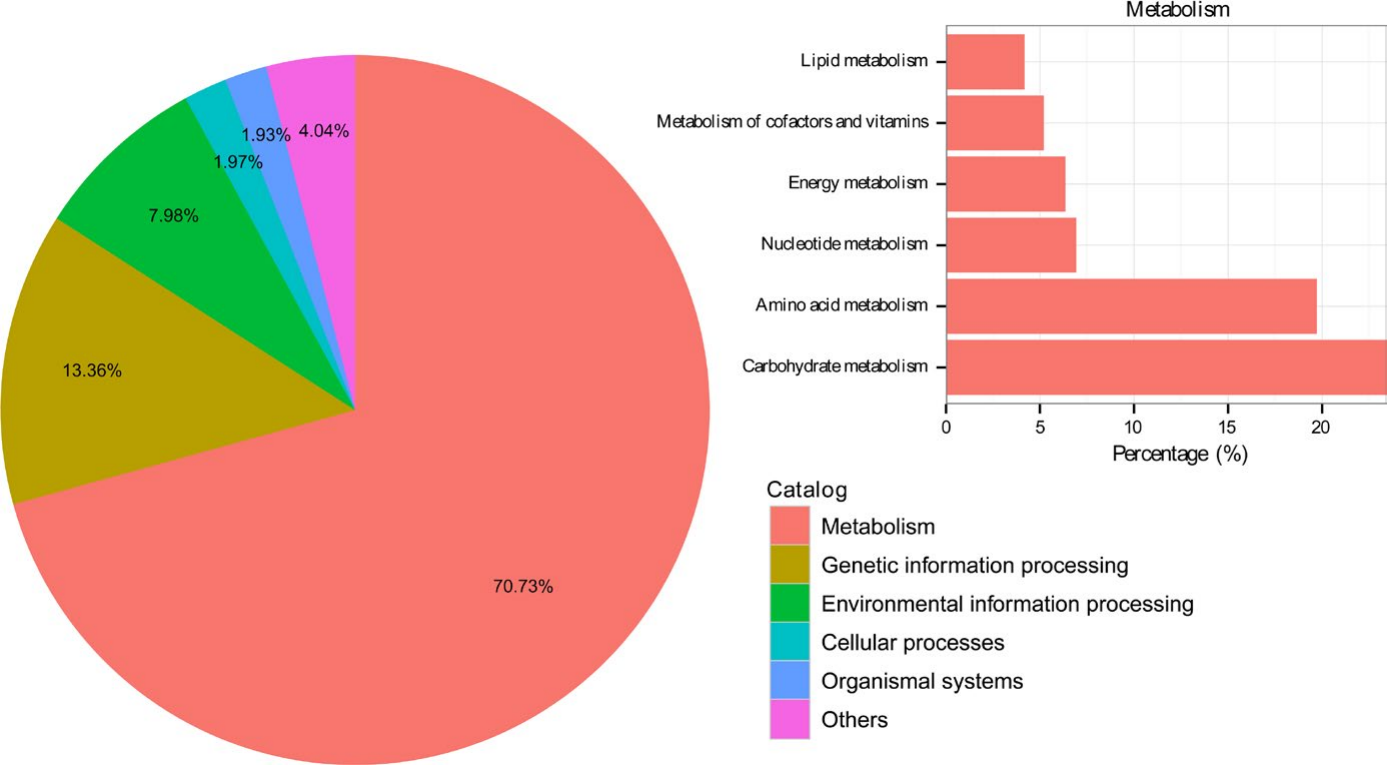
The top five KEGG categories present in the Bar‐headed geese metagenome

To date, almost nothing is known about the dominant metabolic functions of Bar‐headed geese gut microbiota. As shown in Figure 3, the top three functional categories included metabolism (70.73% of all assigned sequences), genetic information processing (13.36%) and environmental information processing (7.98%) in the KEGG database. The additive abundance of these three categories was above 92.06% of all assigned sequences. Within the KEGG categories, matches were separated into different subcategories (Table S5). Most of the sequences in the subcategory carbohydrate metabolism (23.45%) shared homologies to known genes involved in nucleotide sugars (7,571 sequences), starch and sucrose (6,347 sequences), pyruvate (6,228 sequences) and galactose (4,977 sequences). In addition, sequences were homologous to genes responsible for glycolysis/gluconeogenesis (6,349 sequences), the pentose‐phosphate pathway (4,174 sequences) and the citrate cycle (3,128 sequences) (Table S5). The second most of the sequences were assigned in the category amino acid metabolism (19.72%), followed by nucleotide metabolism (6.93%) and energy metabolism (6.35%) (Figure 3). These results indicated a high metabolic versatility of the gut microbiome related to Bar‐headed geese.

## 4 Discussion

The dataset presented in this study is the first to functionally characterize the gut microbial communities of Bar‐headed geese. Taxonomic analyses revealed that a highly complex bacterial community was present in the gut of Bar‐headed geese. Much like for other species of birds, taxonomic data indicated that *Firmicutes*,* Proteobacteria*,* Actinobacteria*, and *Bacteroidetes* were the four most abundant phyla (Dewar et al., 2014; Waite, Deines, & Taylor, 2012; Xenoulis et al., 2010). Comparison of the gut microbiota at the phylum level identified that *Bacteroidetes* were significantly abundant in the AR group compared to the wild group. Members of *Bacteroidetes* possess very large numbers of genes‐encoding carbohydrate active enzymes, which allows them to switch readily between different energy sources in the gut depending on availability, using sophisticated regulatory mechanisms to control gene expression (Thomas, Hehemann, Rebuffet, Czjzek, & Michel, 2011). Therefore, we postulate that the emergence of a large number of *Bacteroidetes* in AR group may contribute to the Bar‐headed goose adaption to the digestion of both wild and artificial food resources. Additionally, the ratio of *Firmicutes* to *Bacteroidetes* was found to be 72‐fold higher in wild group than in AR group. In the human gut microbiome, an increase in fiber content of the dietary regime was associated with a decrease in the ratio of *Firmicutes* to *Bacteroidetes* (De Filippo et al., 2010). Therefore, the lower representation of *Firmicutes* and higher representation of *Bacteroidetes* in the AR group might be attributable to the higher fiber content of the artificial diets.

Through functional profiling we found that artificially reared Bar‐headed geese had higher bacterial gene content related to carbohydrate transport and metabolism, energy metabolism and coenzyme transport and metabolism. Most fragments in which the top BLASTX sequence match showed significant similarity to a *Bacteriodetes* protein in AR group. Therefore, these differences in functional groups are likely attributed to the bacterial gene content from *Bacteroidetes*, which have a wide capacity to use diverse types of polysaccharides (Xu et al., 2007). Excess of these phylum microbiota may therefore confer more efficient extraction of energy from both the natural and artificial food resources. We also observed marked differences between two groups at the KEGG pathway level, “Protein digestion and absorption”, “Glycosphingolipid biosynthesis ‐ ganglio series” and “Lysosome” were enriched in samples from the artificially reared Bar‐headed geese. These results might be associated with increased dietary protein contents and its digestibility. Corn and soybean are the most widely used protein feed in animal husbandry worldwide because of its high protein content, high digestibility and relatively‐balanced amino acid profile. These two food materials were also used in artificially reared Bar‐headed geese diet. The *Bacteroides* was found to be more abundant in those people who preferred to eat high protein (Wu et al., 2011). The increase in proportion of *Bacteroides* in AR group maybe due to the same reason of high dietary protein. While it is still unclear how each of the predicted functional differences observed in our study relate to different dietary contents of Bar‐headed geese, these findings do provide basis for future study.

To build a comprehensive gene catalog of Bar‐headed geese metagenome, we combined de novo assembled clean reads from all samples. An overwhelming majority of KOs belonged to three main families: metabolism, genetic information processing and environmental information processing. These overrepresented metabolic pathways might be related to the energy consumption to fulfill a variety of physiological activities of the host. In fact, avian metabolism is around 60% higher than most mammals (Scanes & Braun, 2012). This high metabolic rate is of course required for the demands of flight. Among the KEGG metabolism subcategories, metabolism of carbohydrate, amino acid, nucleotide and energy were found to be the four most abundant categories in Bar‐headed geese gut metagenome, indicating that the metabolic potential of these gut microbes is highly diverse and versatile. They are well adapted to degrade carbohydrates and amino acids and derivatives. Even though our study cannot provide evidence for direct causal effects between functional differences and the reproductive rate, these findings provide preliminary insight into how metabolic pathways are altered between the wild and artificially reared groups, and this work may help in better understanding of microbial genetic factors that are relevant to the reproduction of Bar‐headed geese.

## 5 Conclusions

In summary, this is the first description of the Bar‐headed goose gut microbial community using a metagenome sequence analysis. Comparative metagenomic analyses identified differences in the structure and function of gut microbial communities between wild and artificially reared Bar‐headed geese. Even though our study cannot provide evidence for direct causal effects, these findings can serve as the foundation for future analyses to examine changes in the compositions and metabolic activities of gut microbiota during the rearing period as well as in response to environmental changes, such as artificial diets and living conditions. As additional metagenomic information is obtained from bird gut communities, this information will also be useful in comparative metagenomic studies to those interested in understanding the genetic network and ecological roles of avian gut microbial populations and for the identification of selective pressures imposed by artificially reared practices on host gut metagenome.

## Conflict of Interest

None declared.
