# Supplementary material for: Metagenomic profiling of gut microbial communities in both wild and artificially reared Bar‐headed goose (Anser indicus)
Source: Microbiologyopen. 2016 Dec 20;6(2):e00429. doi: 10.1002/mbo3.429 (PMC5387313; doi:10.1002/mbo3.429)
Supplement: Supplementary file 1 [file MBO3-6-na-s001.docx]

**Supporting Information**

**for**

**Metagenomic profiling of gut microbial communities in both wild and artificially reared Bar-headed goose (*Anser indicus*)**

Wen Wang^1,2^, Sisi Zheng^2,3^, Kirill Sharshov^4^, Hao Sun^2^, Fang Yang^2^, Xuelian Wang^2^, Laixing Li^2^ & Zhixiong Xiao^1^

^1^Center of Growth, Metabolism and Aging, Key Laboratory of Bio-Resource and Eco-Environment of Ministry of Education, College of Life Sciences and State Key Laboratory of Biotherapy, Sichuan University, Chengdu 610000, China

^2^Key Laboratory of Adaptation and Evolution of Plateau Biota, Northwest Institute of Plateau Biology, Chinese Academy of Sciences, Xi'ning 810008, China

^3^University of Chinese Academy of Sciences, Beijing 100101, China

^4^Research Institute of Experimental and Clinical Medicine, Novosibirsk 630117, Russia

**Table S1.** Raw data before and after standard quality control filters.

|  | Raw | | | | Post Quality Control | | | |
| --- | --- | --- | --- | --- | --- | --- | --- | --- |
| Samples | R1_reads | R1_bases | R2_reads | R2_bases | R1_reads | R1_bases | R2_reads | R2_bases |
| AR_1 | 18,598,811 | 2,343,450,186 | 18,598,811 | 2,343,450,186 | 17,328,973 | 2,050,994,655 | 17,328,973 | 2,127,372,651 |
| AR_2 | 23,166,221 | 2,918,943,846 | 23,166,221 | 2,918,943,846 | 21,519,060 | 2,537,952,973 | 21,519,060 | 2,642,106,234 |
| Wild_1 | 23,097,738 | 2,910,314,988 | 23,097,738 | 2,910,314,988 | 21,422,673 | 2,543,619,247 | 21,422,673 | 2,629,331,309 |
| Wild_2 | 21,574,311 | 2,718,363,186 | 21,574,311 | 2,718,363,186 | 20,168,086 | 2,373,527,041 | 20,168,086 | 2,481,590,404 |
| Total | 86,437,081 | 10,891,072,206 | 86,437,081 | 10,891,072,206 | 80,438,792 | 9,506,093,916 | 80,438,792 | 9,880,400,598 |

**Table S2.** The number of clean reads per sample used for further clear assembly and annotation analysis.

| Samples | Quality_pass_reads | Clean_reads |
| --- | --- | --- |
| AR_1 | 34,657,946 | 7,582,586 |
| AR_2 | 43,038,120 | 39,269,524 |
| Wild_1 | 42,845,346 | 5,019,282 |
| Wild_2 | 40,336,172 | 5,431,620 |
| Total | 160,877,584 | 57,303,012 |

**Table S3.** The number of clean reads matched to COG and KEGG database in each sample.

| Sample | Total clean reads | COG reads | COG genes | KEGG reads | KEGG genes | KEGG pathways |
| --- | --- | --- | --- | --- | --- | --- |
| AR_1 | 7,582,586 | 3,388,539(44.69%) | 3,982 | 2,261,878(29.83%) | 5,174 | 315 |
| AR_2 | 39,269,524 | 13,832,280(35.22%) | 4,277 | 9,101,384(23.18%) | 5,935 | 357 |
| Wild_1 | 5,019,282 | 2,253,735(44.90%) | 3,862 | 1,533,401(30.55%) | 4,798 | 317 |
| Wild_2 | 5,431,620 | 2,104,371(38.74%) | 3,963 | 1,427,603(26.28%) | 4,990 | 343 |

**Table S4.** The top 10 COG genes enriched in phylum *Bacteroidetes* in AR group were found to be distributed in *Firmicutes* and *Proteobacteria* in wild group.

| COG | COG category and description | Top BlastX hit organism | |
| --- | --- | --- | --- |
|  |  | AR | Wild |
| COG0642 | [T], Signal transduction histidine kinase | Bacteroidetes (60.81%) | Firmicutes (48.69%) |
| COG0480 | [J], Translation elongation factors (GTPases) | Bacteroidetes (56.02%) | Firmicutes (81.83%) |
| COG3250 | [G], Beta-galactosidase/beta-glucuronidase | Bacteroidetes (79.11%) | Firmicutes (86.39%) |
| COG0841 | [P], Cation/multidrug efflux pump | Bacteroidetes (83.91%) | Proteobacteria (68.06%) |
| COG2207 | [K], AraC-type DNA-binding domain-containing proteins | Bacteroidetes (72.21%) | Firmicutes (56.81%) |
| COG1472 | [G], Beta-glucosidase-related glycosidases | Bacteroidetes (64.12%) | Firmicutes (74.45%) |
| COG0845 | [M], Membrane-fusion protein | Bacteroidetes (86.54%) | Proteobacteria (79.54%) |
| COG1538 | [M], Outer membrane protein | Bacteroidetes (87.78%) | Proteobacteria (92.67%) |
| COG3119 | [P], Arylsulfatase A and related enzymes | Bacteroidetes (86.37%) | Proteobacteria (44.91%) |
| COG0793 | [M], Periplasmic protease | Bacteroidetes (89.44%) | Proteobacteria (51.35%) |

**Table S5.** Selected KEGG categories (level 1 to 3) present in the Bar-headed geese metagenome.

* Number of matches to respective KOs is given in parenthesis.

[*The Table is provided as separate file (Table S5) for it is too large to integrate*]
